# Molecular Characterization of a New Tetrodotoxin-Binding Protein, Peroxiredoxin-1, from *Takifugu bimaculatus*

**DOI:** 10.3390/ijms23063071

**Published:** 2022-03-12

**Authors:** Kun Qiao, Chunchun Wang, Luqiang Huang, Huimin Feng, Bei Chen, Min Xu, Yongchang Su, Shuji Liu, Nan Pan, Jie Su, Zhiyu Liu

**Affiliations:** 1 Fisheries Research Institute of Fujian, Key Laboratory of Cultivation and High-Value Utilization of Marine Organisms in Fujian Province, Xiamen 361013, China; qiaokun@xmu.edu.cn (K.Q.); chenbeifjfri@foxmail.com (B.C.); xumin@jmu.edu.cn (M.X.); suyongchang@126.com (Y.S.); cute506636@163.com (S.L.); npan01@qub.ac.uk (N.P.); sjscut@126.com (J.S.); 2 College of Life Sciences, Fujian Normal University, Fuzhou 350108, China; qsx20190968@student.fjnu.edu (C.W.); biohlq@fjnu.edu.cn (L.H.); 3 School of Life Science, Xiamen University, Xiamen 361005, China; fenghuimin@stu.xmu.edu.cn

**Keywords:** peroxiredoxin-1, *Takifugu bimaculatus*, radical scavenging, tetrodotoxin-binding protein

## Abstract

Pufferfish are considered a culinary delicacy but require careful preparation to avoid ingestion of the highly toxic tetrodotoxin (TTX), which accumulates in certain tissues. In this study, the tissue distribution of peroxiredoxin-1 from *Takifugu bimaculatus* was investigated. The peroxiredoxin-1 protein was obtained by in vitro recombinant expression and purification. The recombinant protein had a strong ability to scavenge hydroxyl radicals, protect superhelical DNA plasmids from oxidative damage, and protect L929 cells from H_2_O_2_ toxicity through in vitro antioxidant activity. In addition, we verified its ability to bind to tetrodotoxin using surface plasmon resonance techniques. Further, recombinant proteins were found to facilitate the entry of tetrodotoxin into cells. Through these analyses, we identified, for the first time, peroxiredoxin-1 protein from *Takifugu bimaculatus* as a potential novel tetrodotoxin-binding protein. Our findings provide a basis for further exploration of the application of peroxiredoxin-1 protein and the molecular mechanisms of tetrodotoxin enrichment in pufferfish.

## 1. Introduction

*Takifugu bimaculatus* is a carnivorous groundfish that belongs to the Tetraodontiformes and is widely distributed from the South Yellow Sea to the South China Sea. Its delicious, chewy taste and high economic value make this fish popular in Southeast Asian markets [1,2]. *T. bimaculatus* can survive at lower latitudes and can adapt to higher temperatures and salinities as compared to the other three cultural pufferfish species in China; thus, farming production of *T. bimaculatus* has attracted much interest in recent years [3]. Accordingly, research on *T. bimaculatus* has mainly focused on genetic breeding and cultivation techniques. However, pufferfish contain highly toxic tetrodotoxin (TTX), which poses a significant risk to food safety.

TTX is a highly toxic non-protein natural neurotoxin that selectively blocks Na^+^ channels, alters Na^+^ membrane permeability, has a highly specific effect on nerve cell membranes, and possesses unique nerve blocking properties that block excitation transmission between nerves and muscles [4]. The median lethal dose (LD_50_) for TTX given intraperitoneally to mice is 10.7 μg/kg [5]. To date, TTX has been identified in or on the body surface of many vertebrates and invertebrates, as well as in marine microorganisms [6]. Levels of TTX are significantly correlated with species, tissues, and seasons. The tissue distribution of TTX in pufferfish is highly variable between species and the toxicity is correlated with the aquatic environment [7].

Peroxiredoxins (Prxs) are a class of mammalian antioxidants observed in most tissues of various fish species that use active center-reactive cysteines to promote the reduction of H_2_O_2_, organic hydroperoxides, and peroxynitrite to H_2_O, thereby protecting cells and tissues from oxidative and nitrosative stress and serving as ideal H_2_O_2_ scavengers [8,9]. Prxs are also considered the first line of defense against oxidative damage caused by high levels of reactive oxygen species (ROS) [10]. Peroxiredoxin-1 (Prx-1) has been shown to have the highest enzymatic activity among all peroxisomal proteins [11]. Prx-1 is mainly characterized as a regulator of cell proliferation, differentiation, and apoptosis [12]. Prx-1 conserved universal markers of circadian rhythms in a variety of life [13]. In addition, it plays certain roles in many pathological states; for example, prx-1 as an NK enhancing factor A (NKEF-A), has been identified as an erythrocyte cytosolic protein and stimulates NK activity. Thus, prx-1 deletion causes hemolytic anemia, and a shortened lifespan. Furthermore, the malignancies (lymphomas, sarcomas, and carcinomas) are frequently associated with loss of prx-1 expression in heterozygotes, which suggests that this protein functions as a tumor suppressor. Some researchers also pointed out that prx-1 contributed to regulating inflammation [14].

In this study, we identified Tbprx-1 as a novel TTX-binding protein. In recent years, several interesting discoveries have been made regarding TTX-binding proteins in pufferfish. Initially, saxitoxin (STX) and TTX-binding protein (PSTBP) were purified from the plasma of *Takifugu niphobles*. The molecular weight of the purified protein was determined to be 91,000, and the TTX-binding activity of the final fraction was 66% [15]. Subsequently, PSTBP was found in various types of toxic pufferfish [16,17,18,19,20,21,22,23], and was assumed to be a carrier protein to transfer tetrodotoxin among the tissues, particularly liver, ovary, and skin; PSTBPs localize to the dermis surrounding TTX-secreting glands and later transport TTX to the glands [19]. PSTBPs comprise two tandem repeated homologous domains, and the high sequence similarity to tributyltin-binding protein 2 (TBT-bp2) shows that PSTBPs originated from duplications and fusions of pufferfish TBT-bp2s. TBT-bps are known as fish alpha 1-acid glycoprotein (AGP)-like lipocalin proteins that bind to tributyltin, which is highly toxic to aquatic organisms [21]. In addition, TTX-binding protein was recently isolated and purified from ovarian extracts of *Takifugu pardalis* and identified as vitellogenin, a precursor of the major egg yolk protein, which is synthesized primarily in the liver, distributed throughout the body by the systemic circulation, and then carried to the ovary. Vitellogenins bind with specific receptors anchored in the oocyte plasma membrane and internalized with their vitellogenin ligands during passage through the ovary. Vitellogenin, cleaved into phosvitin, lipovitellins (I and II), and the vWF type D domain, are stored in early endosomes in oocytes [24]. A subsequent study cloned a cDNA fragment of the vitellogenin gene containing the VWD domain from *Takifugu flavidus* (TfVWD), and determined that key amino acids (namely, Val115, ASP116, Val117, and Lys122) in TfVWD mediate its binding to tetrodotoxin by silico structural and docking analyses of the predicted protein. The expression profiling of TfVWD across different tissues and developmental stages indicates that its distribution patterns mirror those of tetrodotoxin, suggesting that TfVWD may be involved in tetrodotoxin transport in pufferfish [25].

In this study, we investigated the tissue distribution of the *prx-1* gene in *T. bimaculatus* in vivo, and obtained recombinant Tbprx-1 for function evaluations. Molecular docking predicted the binding sites to TTX and the binding ability was evaluated by surface plasmon resonance (SPR) technique. This study will provide a theoretical basis and reference for further studies on the migration and enrichment of TTX in puffer fish.

## 2. Results

### 2.1. Tissue Distribution of Tbprx-1 mRNA

**Figure 1:**
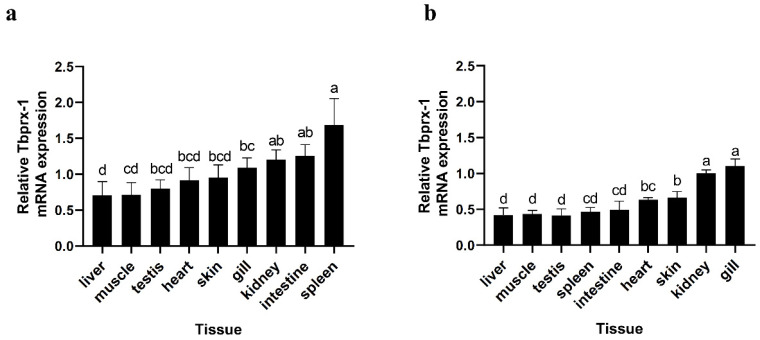
Expression of *peroxiredoxin-1* in various tissues and organs for (**a**) immature and (**b**) mature *T. bimaculatus*. The housekeeping gene was *18S*. Bars show the standard errors of mean values. The results were analyzed using an independent-samples *t*-test. Different letters (a,b,c,d) indicate significant differences in the same group (*p* < 0.05); the same letter indicates no significant difference (*p* > 0.05).

A quantitative polymerase chain reaction (qPCR) method was used to investigate the distribution of *prx-1* transcripts among the tissues of immature and mature *T. bimaculatus* (Figure 1). In immature samples, the spleen had the highest expression; the gill, intestine, and kidney had moderate expression levels; and the liver, muscle, testis, heart, and skin had the lowest expression levels. In contrast, in mature samples, expression was highest in the gill and kidney; moderate in the heart and skin; and lowest in the liver, muscle, testis, spleen, and intestine.

### 2.2. Characterization and Phylogenetic Analysis of the Tbprx-1 Gene

**Figure 2:**
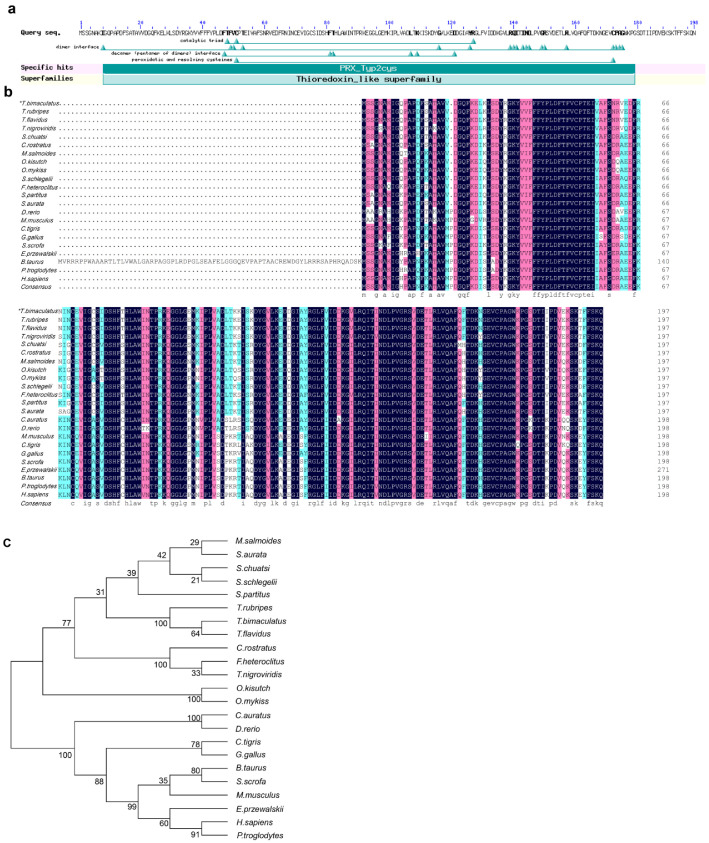
(**a**) Structural domain prediction for Tbprx-1. (**b**) Alignment of prx-1 in *Takifugu* *bimaculatus* with those from other species. Multiple sequence alignment of Tbprx-1 (TNM85992.1) and *T. flavidus* (TWW58563.1), *Takifugu rubripes* (XP_003972530.1), *Tetraodon nigroviridis* (ABC59169.1), *Siniperca chuatsi* (XP_044045544.1), *Chelmon rostratus* (XP_041819927.1), *Micropterus salmoides* (XP_038570209.1), *Gallus* (NP_001258861.1), *Homo sapiens* (NP_001189360.1), *Danio rerio* (NP_001013489.2), *Bos taurus* (NP_776856.1), *Pan troglodytes* (XP_001156568.1), *Sus scrofa* (XP_020952401.1), *Crotalus tigris* (XP_039205522.1), *Equus przewalskii* (XP_008519794.1), *Oncorhynchus* *kisutch* (XP_020309895.2), *Oncorhynchus mykiss* (XP_021412501.2), *Carassius auratus* (XP_026051992.1), *Sebastes schlegelii* (QGN18756.1), *Fundulus heteroclitus* (XP_012725625.2), *Stegastes partitus* (XP_008276119.1), *Mus musculus* (NP_035164.1) and *Sparus aurata* (XP_030284090.1). * indicate species in this study. (**c**) Phylogenetic tree constructed based on the deduced amino acid sequence of Tbprx1 and other genes of the peroxiredoxin (PRX) superfamily using the neighbor-joining method.

Structure prediction showed that the Tbprx-1 protein sequence contains two highly conserved cysteines (Cys51 and Cys172; Figure 2a). Tbprx-1 encodes 198 amino acids with a molecular weight of 22.08 kDa and a predicted isoelectric point of 5.47. The signal peptide was not detected in the present gene sequence. The results of amino acid sequence comparison with other known species are shown in Figure 2b. To elucidate the molecular evolutionary relationships, a phylogenetic tree was constructed by comparing *T. bimaculatus* with the Tbprx-1 gene from different species. The tree (Figure 2c) depicts evolutionary relationships based on the similarity of Tbprx-1 proteins from different species; the results show that the Tbprx-1 protein is 99.5% homologous to the prx-1 protein of *T. flavidus*, which is closer than that of other species.

### 2.3. Recombinant Expression Vector Construction and Recombinant Tbprx-1 Protein Purification

**Figure 3:**
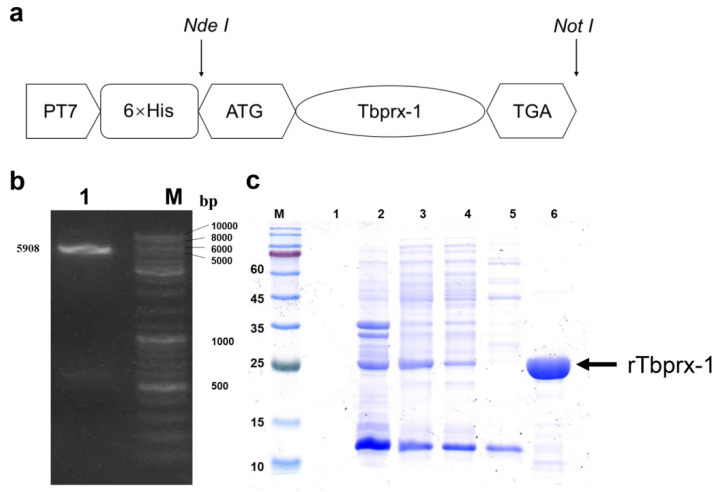
Construction of the recombinant expression vector. (**a**) Structural map of the pET-28a(+)- *Tbprx-1* recombinant plasmid vector. (**b**) pET-28a(+) plasmid map after double enzymatic digestion. Lane M: DNA Ladder Mix; lane 1: after enzymatic cleavage of the vector. (**c**) M: molecular weight marker (Blue Plus IV Protein Marker [10–180 kDa], TransGen Biotech, Beijing, China). 1, recombinant *E. coli* BL21 (DE3) strain without IPTG; 2, lysed *E. coli* pellets after sonication; 3, lysate soluble fraction from *E. coli* induced with IPTG; 4, proteins that did not bind to Ni-NTA resin; 5, protein eluted from Ni-NTA resin with 70 mM imidazole; 6, purified (His) 6-prx-1 fusion protein obtained after eluting with 500 mM imidazole.

The expression vector used in the experiment contained a T7 lac promoter that induced the secretion of the target gene. The six histidine residues fused at the C-terminus enabled the target protein to be purified using 6× His-Tag affinity chromatography. Figure 3a shows the construction of the pET-28a(+)-*prx-1* expression vector. The amplified *Tbprx-1* target fragment was double digested with the restriction endonucleases NdeI and NotI to obtain a gene fragment that could be ligated with pET-28a(+) containing the same sticky ends. The digested pET-28a(+) plasmid was loaded into a 0.8% (*w*/*v*) agarose gel and subjected to electrophoresis in TAE to measure the cleavage efficiency (Figure 3b).

Tbprx-1 was induced with 0.2 mM isopropyl-beta-D-thiogalactopyranoside (IPTG) and sonication. Fractions were assessed for recombinant Tbprx-1 purity by sodium dodecyl sulfate-polyacrylamide gel electrophoresis (SDS-PAGE) and Coomassie blue staining. The recombinant *E. coli* BL21 strain without induction with IPTG shows no band. A fusion protein Tbprx-1 was highly soluble as shown in Figure 3c. Tbprx-1 protein was purified by affinity chromatography using a HisTrap FF Crude column and AKTA Prime system (both GE Healthcare). Unspecific binding proteins and a little quantity of prx-1 were washed with 70 mM imidazole of the binding buffer. Thereafter pure prx-1 was eluted with 500 mM imidazole, which appeared as a single band of 22.08 kDa (Figure 3c).

### 2.4. Radical Scavenging Ability of rTbprx-1

**Figure 4:**
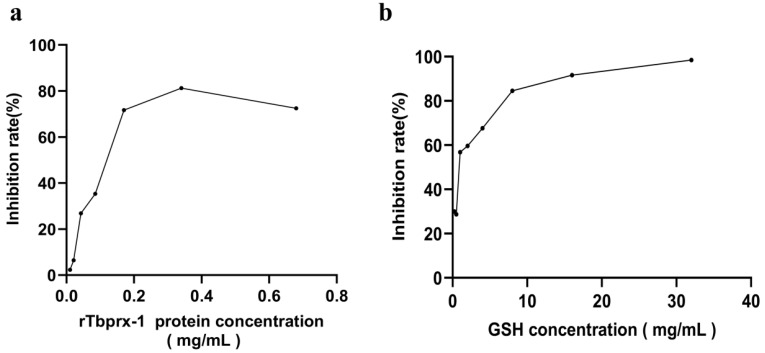
Comparison of hydroxyl free radical scavenging abilities of rTbprx-1 and glutathione (GSH). (**a**) Hydroxyl free radical scavenging ability of the rTbprx-1 expression product. (**b**) Hydroxyl free radical scavenging ability of GSH.

rTbprx-1 had good hydroxyl free radical scavenging ability (Figure 4). The 50% hydroxyl free radical clearance rate for rTbprx-1 was 0.12 mg/mL. When glutathione (GSH) was used as a positive control, the GSH concentration associated with a 50% hydroxyl free radical clearance rate was 0.95 mg/mL. These findings showed that the rTbprx-1 protein possessed a strong hydroxyl free radical scavenging ability.

### 2.5. In Vitro Supercoiled DNA Protection Activity of rTbprx-1

**Figure 5:**
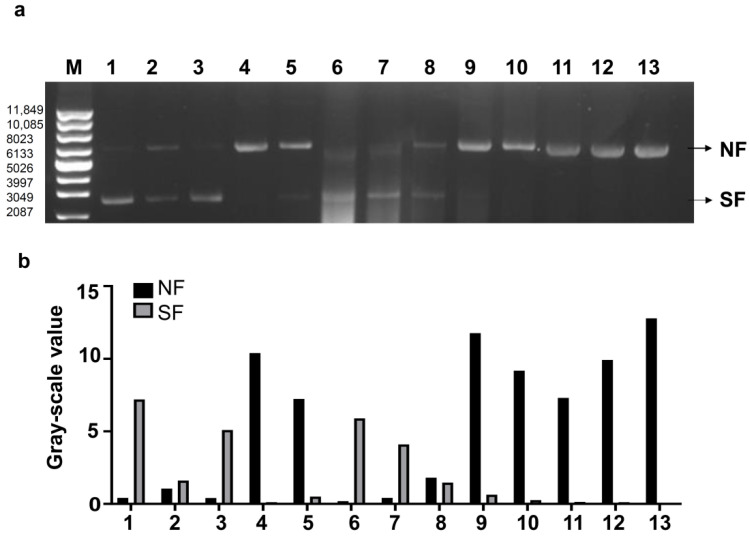
Potential of rTbprx-1 to protect supercoiled DNA from cleavage in a MCO system using DNA nicking assays. (**a**) Protection of supercoiled DNA cleavage by rTbprx-1. (**b**) Gray-scale value of nicked and supercoiled DNA from different lanes in (**a**). M: Supercoiled DNA Ladder Marker. lane 1: pUC18 DNA in water, incubated at 37 °C for 2.5 h; lane 2: pUC18 DNA incubated with 2 mM DTT only; lane 3: pUC18 DNA incubated with 0.2 mM FeCl_3_ only; lane 4: pUC18 DNA in the MCO reaction mixture; lane 5: pUC18 DNA in the MCO reaction mixture with bovine serum albumin (0.5 mg/mL); lanes 6–13: pUC 18 DNA in the MCO reaction mixture with varying concentrations of rTbprx-1 (0.9, 0.45, 0.225, 0.1125, 0.05625, 0.028125, 0.0140625, 0.00703125 mg/mL). NF: nicked form; SF: supercoiled form.

Next, we evaluated the ability of rTbprx-1 to protect in vitro supercoiled DNA. The supercoiled DNA PUC18 is 2686 bp in size in its normal form; following attack by hydroxyl radicals produced by the metal-catalyzed oxidation (MCO) system, the DNA is converted from the superhelical form to the nickel form. The degree of DNA damage is indicated by a pattern of bands with different mobility changes resulting from the electrophoretic breakdown of the two forms of DNA in the gel. In the absence of rTbprx-1, the DNA showed the most damage, whereas adding rTbprx-1 to the MCO system reduced the smear produced by hydroxyl radical-dependent DNA damage in a concentration-dependent manner. The strongest DNA protection was achieved when the protein concentration was 0.9 mg/mL (Figure 5).

### 2.6. Protective Effects of rTbprx-1 against H_2_O_2_-Induced Cellular Damage

**Figure 6:**
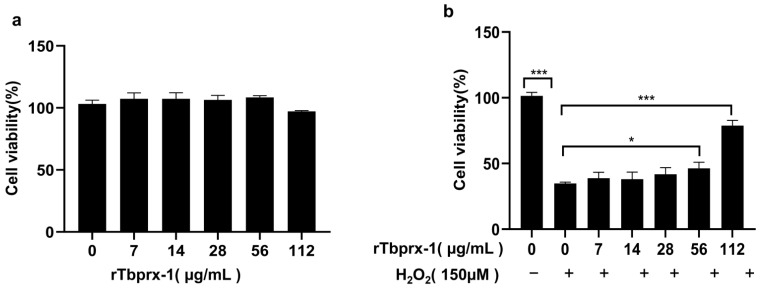
(**a**) Cell viability after treatment of L929 cells with rTbprx-1 (0–112 µg/mL). (**b**) Effects of rTbprx-1 on protection against H_2_O_2_-induced cell death. Cells were treated with 150 µM H_2_O_2_ and different concentrations of rTbprx-1 (7–112 µg/mL), and cell viability was measured using MTS/PMS assays. The results were analyzed using independent-samples t-tests. * *p* < 0.05, *** *p* < 0.001 compared with the control.

To determine whether rTbprx-1 protein reduced cell viability, L929 cells were incubated with purified rTbprx-1 protein for 24 h, and 3-(4,5-dimethylthiazol-2-yl)-5-(3-carboxymethoxyphenyl)-2-(4-sulfonyl)-2 H-tetrazolium/phenazine methosulfate (MTS/PMS) assays were performed. As shown in Figure 6, the protein had no significant effect on cell viability in the concentration range of 0–112 µg/mL as compared with that in untreated cells (Figure 6a). We further evaluated the effects of rTbprx-1 on protection against H_2_O_2_-induced cell damage according to the method described by Qiao [26]. Our results showed that L929 cell viability increased as the protein concentration increased. The viability of cells treated with 56 and 112 µg/mL rTbprx-1 was significantly higher than that in untreated cells (Figure 6b). Thus, the rTbprx-1 protein protected L929 cells from peroxidative damage induced by H_2_O_2_.

### 2.7. Protein Modeling and Molecular Docking

**Figure 7:**
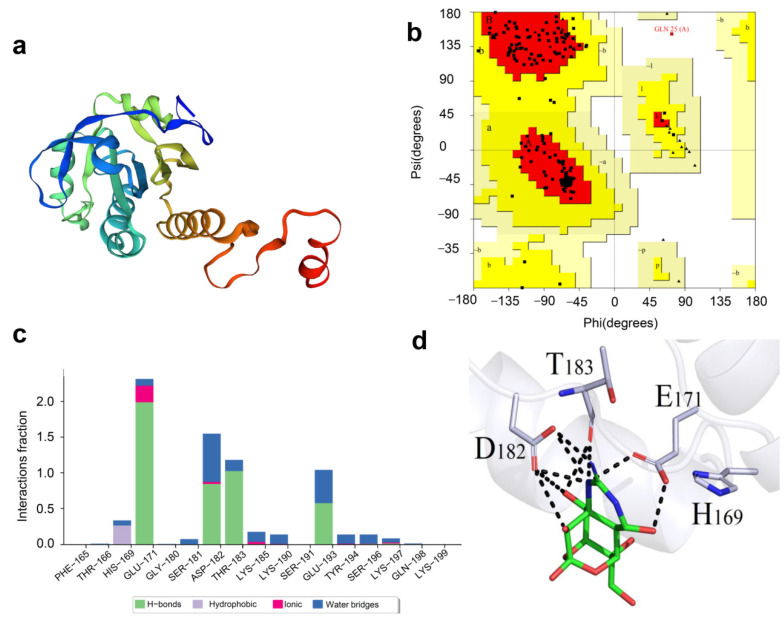
Protein modeling of Tbprx-1 and molecular docking of Tbprx-1 and tetrodotoxin (TTX). (**a**) Diagram of the homology modeling structure of Tbprx-1. (**b**) Ramachandran plot of Tbprx-1. (**c**) Interaction fractions of individual amino acid residue in Tbprx-1. (**d**) Binding model depicted in 3D, with green denoting the ligand and gray rods denoting amino acid residues around the binding pocket. The backbone of the receptor is indicated by a light gray ribbon. The black dashed line indicates the hydrogen bond between the ligand and the receptor.

The homology modeling structure is shown in Figure 7a, and the Ramachandran plot of the best modeling structure is shown in Figure 7b. We selected 2z9s as the template with 87.94% sequence identity; 89.9% of amino acid residues were located in the favored region and 9.5% in allowed regions, with only 0.6% being outliers, indicating the feasibility of the modeled 3D structure. The docking (binding energy) score of TTX to the ab initio predicted protein was −5.81 kcal/mol. The putative binding pocket comprised PRO-581, SER-582, ASN-583, SER-584, THR-649, LEU-650, VAL-651, THR-652, HIS-676, VAL-678, PRO-680, GLU-681, LEU-706, GLY-707, ILE-709, LEU-710, ARG-711, TYR-718, LEU-720, ARG-856, SER-885, GLU-887, GLU-888, GLN-890, and ARG-925. Molecular dynamics simulations were performed using the Desmond module of the Schrodinger package, and amino acids GLU171, ASP182, THR183, and GLU193 were continuously involved in the interaction, with GLU171, ASP182, THR183, and GLU193 contributing more to the formation of hydrogen bonds.

### 2.8. SPR Measurement of the Binding Activity of TTX and rTbprx-1

**Figure 8:**
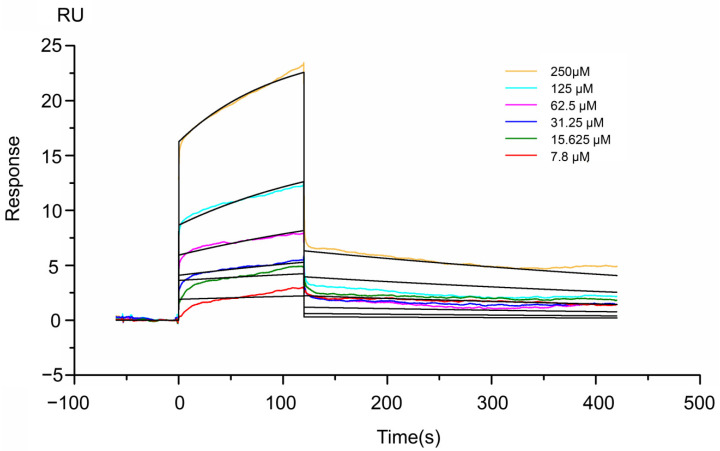
Binding activity of rTbprx-1 with tetrodotoxin (TTX). Steady—state fitting to obtain the KD value of rTbprx-1.

Next, we used SPR to evaluate binding of rTbprx-1 with TTX. The results showed the response value for rTbprx-1 and TTX binding was 10,935.6 RU, and the equilibrium dissociation constant (KD) for rTbprx-1 and TTX was calculated using 4.10 × 10^−5^ M kinetics. (Figure 8).

### 2.9. Effects of rTbprx-1 on TTX Entry into Epithelioma Papulosum Cyprini (EPC) Cells

**Figure 9:**
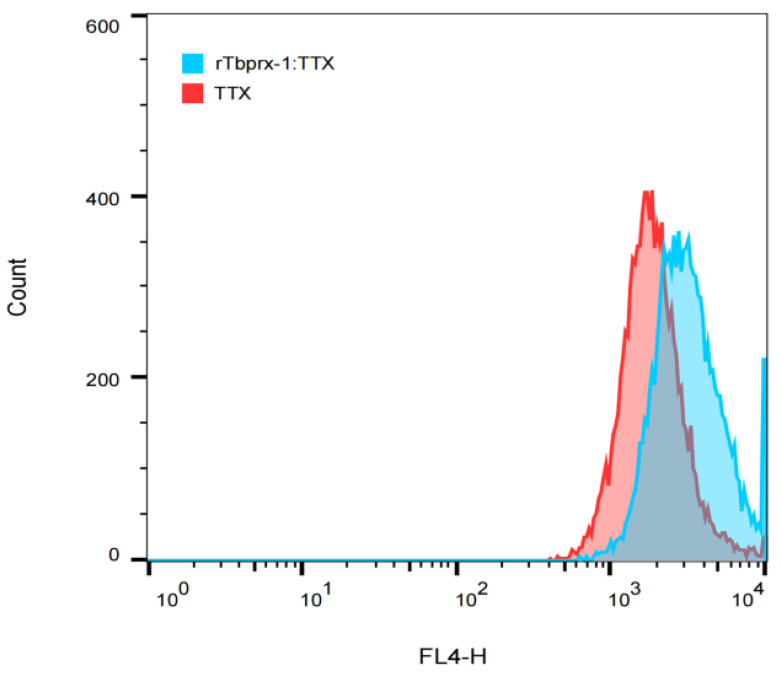
Effects of rTbprx-1 on TTX entry into Epithelioma Papulosum Cyprini (EPC) cells.

The effect of rTbprx-1 on the entry of TTX into EPC cells was examined using flow cytometry as shown in Figure 9. The EPC cells supplemented with rTbprx-1 showed significantly more TTX compared with the control group (Figure 9).

## 3. Discussion

In this study, we investigated the expression characteristics of the *Tbprx-1* gene and the active function of the protein in *T. bimaculatus*. In addition to its good antioxidant activity, a new function of prx-1, which can bind to TTX, was identified by molecular docking and SPR techniques.

The distribution of mRNA expression and protein content of *Tbprx-1* was evaluated. As differential expression of genes in different tissues suggests a direct or indirect relationship between these genes’ functions and these tissues, the relatively high expression level of immune-related tissues suggests that *Tbprx-1* is involved in the immune system in this species. Wang et al. [27] showed that *prx-1* mRNA is most highly expressed in the spleen, intestines, kidney, and gills of the golden pompano. This is consistent with our results and suggests that *Tbprx-1* could play diverse roles in *T. bimaculatus*. The intestine is constantly exposed to ROS generated by the luminal contents (i.e., oxidized food, salivary oxidants) because of its vulnerability to oxidative stress [8]. Multiple sequence alignment results show that the protein was conserved in the evolution of various species. Prx-1 is a typical member of the 2-Cys prx subfamily and is found primarily in the cytoplasm, endoplasmic reticulum, mitochondria, and peroxisomes of cells [11]. Functional domain prediction shows that typical 2-cys prx interacts through beta strands at one edge of the monomer (B-type interface) to form the functional homodimer, and uses an A-type interface (similar to the dimeric interface in atypical 2-cys prx and prx5) at the opposite end of the monomer to form the stable decameric (pentamer of dimers) structure. Furthermore, the N-terminal Cys51 (peroxidatic cysteine = C_P_) in prx-1 forms an intermolecular disulfide bond with another conserved C-terminal Cys172 (resolving cysteine = C_R_) residue and is oxidized to cysteine-sulfonic acid (Cys52-SOH) in the thioredoxin-reducing protein system, which is equivalent to detoxifying peroxides [28]. Thus, prx-1 has been shown to have the highest enzymatic activity among all peroxisomal proteins [29]. These two conservative sites have been found in prx-1 subunits from other species, such as *Lateolabrax japonicus* [30], *Trachinotus ovatus* [27], and *Anoplopoma fimbria* [31]. Interestingly, based on the analysis of amino acid sequence differences of the Tbprx-1 in different species and docking with TTX, GLU193 in the prx-1 was unique in fish and distinct from other species (Figure 2b). This finding suggests that GLU193 may be the key amino acid mediating Tbprx-1 binding to the toxin.

In this study, we obtained recombinant prx-1 from *T. bimaculatus*, which showed a single band at 22 kDa. To obtain the soluble expressed recombinant product, an expression vector pET-28a(+) was used in this study, wherein the rTbprx-1 protein proved to be stable and a highly soluble partner to fuse with the target protein. As expected, the resulting rTbprx-1 was highly expressed in *E. coli* and the soluble component was obtained as shown in Figure 3. The result showed that the rTbprx-1 is highly soluble, most of the rTbprx-1 existed in the sonicated supernatant rather than in inclusion bodies. The recombinant product can be easily purified through immobilized metal ion affinity chromatography. Approximately 20 mg pure rTbprx-1 was obtained from 500 mL of culture medium and 97% purity was achieved by washing and elution with a gradient of imidazole. To assess the ROS-scavenging ability of rTbprx-1, we detected the ability of rTbprx-1 in scavenging hydroxyl free radicals and performed MCO DNA protection assays. The MCO system generates hydroxyl radicals that cause the sugar-phosphate backbone of supercoiled DNA to be cleaved into a nicked form, whereas the reducing agent, DTT, acts as an electron donor to facilitate DNA cleavage. Upon comparison with GSH (which is typically used as a scavenger positive control [32]), the purified rTbprx-1 was found to possess a strong ability to scavenge hydroxyl free radicals. The protection of superhelical DNA by rTbprx-1 is further indication of its antioxidant capacity, and our electropherogram shows slightly less protection compared with the previous research, probably owing to the different activity of rTbprx-1 in different species. The dragging bands that appear in the electropherogram may be caused by interference from proteins. In this evaluation of cell viability of the protein in L929 mouse fibroblast cells, the highest concentration has cytotoxic effects and inhibits cell growth. H_2_O_2_, a stable ROS, has inhibited the growth of L929 cells and results in their apoptosis [33]. Our results showed that rTbprx-1 has a protective effect against H_2_O_2_-induced cell damage. Similarly, Madusanka et al. [34] conducted WST-1 assays to examine FHM cell viability following oxidative cell damage. FHM cells were transfected with pcDNA3.1(+)/SsPrdx1 or pcDNA3.1 (+); compared with untransfected cells, pcDNA3.1 (+)/SsPrdx1 transfected cells displayed significantly higher cell viability than those transfected with pcDNA3.1 (+) or untransfected controls. H_2_O_2_ can cause excessive ROS accumulation. During catalysis, Prxs conserves C_P_ as the site of oxidation to sulfenic acid (C_P_-SOH) by H_2_O_2_, which then forms an inter- or intramolecular disulfide bond with a C_R_. Finally, the disulfides are reduced by cellular thiodisulfide exchange mechanisms to recover C_P_ and C_R_ [35].

Biomolecular interaction analysis using SPR technology was performed on a Biacore T200 system. According to the experimental data, Ka (1/M*S) is 35.74, Kd (1/S) is 0.001467, and the binding kinetics of rTbprx-1 and TTX is characterized by rapid binding/fast dissociation. The affinity shows the binding strength between the two molecules, which is represented by KD. The KD value of rTbprx-1 bind TTX is 4.10 × 10^−5^ M, and the rTfVWD protein has been reported to bind TTX with a KD value of 2.92 × 10^−3^ M, whose affinity is lower than rTbprx-1 [25]. The protein-small molecule binding affinity constant or KD value is usually between 10^−3^ to 10^−6^ M, indicating there is an interaction between rTbprx-1 and TTX. To further study the effect of rTbprx-1 on TTX transport, we co- EPC cells with rTbprx-1 and TTX, and found that rTbprx-1 can promote the entry of TTX into cells. Based on the kinetic diagram of SPR, our experimental sensorgram is of the “fast up and fast down” type (fast binding and fast dissociation), suggesting that the combination of TTX and rTbprx-1 is instantaneous, and dissociates under specific conditions, which is conducive to the transport of TTX. Further investigation is needed to elucidate how rTbprx-1 transports TTX in vivo and which transcription factors are involved in regulation.

## 4. Materials and Methods

### 4.1. Animals and Tissue Collection

*T. bimaculatus* was purchased from the Takifugu Breeding Farm in Fujian Province. Three immature and three mature puffer fish were collected and histologically observed. The immature ones were one-year-old fish (16.3 ± 0.90 cm in length and 108.0 ± 12.2 g in weight); the mature ones were two-year-old fish (19.1 ± 0.74 cm in length and 187.0 ± 12.3 g in weight). After induction of anesthesia with 0.01% Eugenol cement(Medical Equipment Factory of Shanghai Medical Instruments Co., Ltd., Shanghai, China), fish were dissected, and nine tissue types (kidney, intestine, spleen, liver, heart, gill, skin, muscle, and testis) were collected from each fish. Approximately 100 mg of each tissue was cut into small pieces (<0.5 cm), and the samples were immediately placed in RNAstore Reagent (Tiangen, Beijing, China) and stored at −20 °C overnight or at 4 °C for subsequent transcriptional expression studies.

### 4.2. qPCR Analysis of rTbprx-1 Expression in T. bimaculatus

Total RNA was extracted using an RNAprep Pure Tissue Kit (cat. no. DP431; Tiangen, Beijing, China). After determining the total RNA concentration using an Infinite200 Pro microplate reader (TECAN, Männedorf, Switzerland), 4 µg total RNA was extracted from each tissue, and cDNA was synthesized by reverse transcription using Evo M-MLV Reverse Transcription Reagent Premix (for qPCR; Accurate Biotechnology, Hunan, China) according to the manufacturer’s instructions. The qPCR was conducted in a final volume of 20 µL, containing 10 ng total RNA, 10 pmol specific primers (*prx-1* forward-[F]: 5′-GAGGTCATTGGCTGCTCCAT-3′ and *prx-1* reverse-[R]: 5′-CTTCCTTCAGGACGCCGTAG-3′), and 10 µL FastStart Universal SYBR Green Master (Roche). The reaction conditions were as follows: pre-incubation at 95 °C for 600 s; followed by 45 cycles of denaturation at 95 °C for 10 s, annealing at 60 °C for 10 s, and extension at 72 °C for 10 s; melting (95 °C for 10 s, 60 °C for 10 s, and 97 °C for 1 s). Primers for *18S* (q*18S*-F: 5′-TCCGATAACGAACGAGACTCCG-3′; q*18S*-R: 5′-CATCTAAGGGCATCACAGACCTGT-3′) were designed to amplify the internal reference gene. The qPCR data were calculated using the 2^−∆∆CT^ method. Melt-curve analysis was performed to analyze the specificity of PCR products.

### 4.3. Construction of the rTbprx-1 Vector

The Tb*prx-1* gene was synthesized, and the tag sequence was added at the N-terminal end. The target gene was amplified, double digested, and ligated between NdeI and XhoI of the pET-28a(+) vector. The product was transformed into *E. coli* BL21 (DE3) pLysS (Vazyme, Nanjing, China), inoculated onto LB (Luria-Bertani) agar plates containing 250 µL/mL kanamycin, and incubated at 37 °C. Positive clones were obtained, and the plasmid was confirmed by DNA sequencing through Sangon Biotech Co., Ltd. (Shanghai, China).

### 4.4. Prokaryotic Expression and Purification of the pET28a-Tbprx-1 Vector

A single colony was grown in 10 mL LB medium (10 g/L tryptone, 5 g/L yeast extract, 10 g/L NaCl, pH 7.4) and cultured at 37 °C with shaking at 180 rpm until an OD_600_ value of 2.0–6.0 was obtained. This bacterial broth was inoculated into the LB liquid medium containing kanamycin at a ratio of 1:50, and the cells were then incubated at 37 °C with shaking at 180 rpm until an OD_600_ value of 0.5 was obtained. Next, 0.2 mM IPTG was added, and the cultures were incubated at 37 °C with shaking at 180 rpm for 5 h to induce expression. The culture was centrifuged at 6000× *g* and 4 °C for 15 min, washed twice with phosphate-buffered saline, resuspended, sonicated, and lysed until the liquid was brightly colored. The cultures were then centrifuged at 12,000× *g* and 4 °C for 10 min, and the supernatant was collected. SDS-PAGE was then used to analyze the expression level of the target protein. The supernatant was filtered through a 0.45-μm filter membrane before purification.

Purification was conducted using an AKTA Purifier 100 (GE Healthcare Life Sciences, Waukesha, WI, USA) and immobilized metal affinity chromatography. Three to five column volumes of MilliQ water were used to wash the prepacked column (HisTrapTM FF Crude 5 mL; GE Healthcare Life Sciences, Uppsala, Sweden), and 8–10 column volumes of equilibration buffer (20 mM phosphate buffer, 50 mM NaCl, 70 mM imidazole, pH 7.4) were used to equilibrate the column. The filtered supernatant was loaded onto the column at 1 mL/min, and the eluate was collected. Next, 5–8 column volumes of equilibration buffer were used to wash away unbound protein from the column, after which elution buffer (20 mM phosphate buffer, 50 mM NaCl, 500 mM imidazole, pH 7.4) was used to elute the protein. The elution peaks of the protein were collected, and small amounts were validated using SDS-PAGE by the PAGE Gel Fast Preparation kit (EpiZyme, Shanghai, China).

### 4.5. Radical Scavenging Ability of rTbprx-1

The hydroxyl radical scavenging activity of rTbprx-1 was detected using theHydroxyl Radical Assay Kit(A018; Nanjing Jiancheng Bioengineering Institute, Nanjing, China). Glutathione was used as the positive control experimental group; blank tubes, control tubes, and test tubes were set up to form three groups each for each reaction. After adding the reagent solution in a 37 °C water bath for 1 min, 2 mL of color developer prepared in advance was added and the mixture was protected from light for 10 min. The OD_550_ value was measured using an enzyme marker. The hydroxyl radical inhibition rate was calculated according to the instructions.

### 4.6. MCO Protection Assay

rTbprx-1 can protect superhelical DNA from DTT/Fe^3+^/O_2_ mediated damage, as assessed by in vitro MCO assays [36,37]. Briefly, the 50-μL reaction system contained 0.2 mMFecl3, 2 mM DTT, and different concentrations of rTbprx-1. Samples were incubated for 30 min at 37 °C, and 500 ng pUC18 superhelical DNA plasmid was then added for another 2 h. DNA degradation was evaluated using 8% agarose gel electrophoresis. Reactions with 0.5 mg/mL bovine serum albumin under the same conditions were used as controls.

### 4.7. Measurement of the Antioxidant Activity of rTbprx-1

The effects of rTbprx-1 on the proliferation of L929 mouse fibroblasts were detected using MTS/PMS assays. Briefly, L929 cells were treated with different concentrations of rTbprx-1 (0, 7, 14, 28, 56, 112 µg/mL) and then cultured at 37 °C in an atmosphere containing 5% CO_2_ for 24 h. MTS/PMS cell proliferation working solution was added, and the cells were incubated in the dark for 0.75–2 h. Absorbance was measured at 490 nm using a microplate reader.

In the cell oxidative damage experiment, L929 cells were treated with 150 µM H_2_O_2_ and different concentrations of rTbprx-1 protein. Untreated cells were used as a blank control. The cells were incubated at 37 °C in an atmosphere containing 5% CO_2_ for 3 h before the MTS/PMS assay, and assays were performed as described above.

### 4.8. Protein Homologous Modeling and Molecular Docking

The SWISS-MODEL, 2z9s was selected as a template for homology modeling and protein structural plausibility assessment SAVES v5.0 server (University of California, Los Angeles, CA, USA)was used to assess the structure.

Molecular docking operations were performed using the AutoDock program, Version 4.02 (Scripps Research Insititute, La Jolla, CA, USA) in two steps: the first was to initially identify the tetrodotoxin-binding sites by blind docking; the second was to predict the mode of action of tetrodotoxin at each protein binding site by exact docking.

### 4.9. SPR Measurement of the Binding Activity of TTX and rTbprx-1

We diluted the rTbprx-1 to 80 μg/mL in immobilization buffer 10 mM sodium acetate (pH 4.0). The activator was prepared by mixing 400 mM EDC and 100 mM NHS (GE) immediately before injection. The CM5 sensor chip was activated for 420 s with the mixture at a flow rate of 10 μL/min. Then, 80 μg/mL of rTbprx-1 in immobilization buffer of 10 mM sodium acetate (pH 4.0) was injected into the sample channel (Fc4); the immobilization level was 10,935.6 RU. The chip was deactivated by 1 M ethanolamine hydrochloride (GE)

We diluted TTX with the same running buffer to six concentrations. TTX was injected to Fc3-Fc4 of the channel at a flow rate of 30 μL/min for an association phase of 120 s, followed by 300 s of dissociation. The association and dissociation processes were all performed in the running buffer. We then repeated the six cycles of interaction analysis according to analyte concentrations in ascending order. After each cycle of interaction analysis, the sensor chip surface regenerated completely; we used 10 mM Glycine-HCl as an injection buffer at a flow rate of 30 μL/min for 30 s to remove the ligand and any bound analyte.

### 4.10. Effects of rTbprx-1 on TTX Entry into EPC Cells

Epithelioma Papulosum Cyprini (EPC) cells were seeded in 6-well plates at a density of 3 × 10^5^ cells and incubated in MEM containing 10% FBS and 1% penicillin-streptomycin at 28 °C with 5% CO_2_. rTbprx-1 (112 μg/mL) in culture medium was co-incubated with EPC cells for 24 h. Then, 0.07 μg of TTX labelled with cy5 fluorescent dye marker was added and the plates were wrapped with tin foil to prevent fluorescence burst. The wrapped plates were further incubated at 28 °C for 12 h. The cells were then washed twice with DPBS, scraped and collected into EP tubes, centrifuged, and the supernatant was discarded. Finally, the cells were resuspended in 500 μL DPBS, and the fluorescence signal of cy5-TTX in the cells was detected on a BD FACSCalibur flow cytometer (BD Biosciences). The parameters were adjusted with those of negative cells (cells without any treatment) to detect the number of cells in the FL4 channel for cy5-TTX added alone and the number of cells in the FL4 channel for rTbprx-1 and cy5-TTX added; data were analyzed using Flowjo v.10 sofware (FlowJo LLC, Ashland, OR, USA).

### 4.11. Statistical Analysis

One-way analysis of variance (ANOVA) followed by Tukey’s tests and independent-samples t-tests were performed to compare differences using the SPSS software, version 24.0 (IBM, Armonk, NY, USA). All data are expressed as means ± standard errors, and results at *p* < 0.05 were considered significant.

## 5. Conclusions

We expressed a recombinant prx-1 protein from *Takifugu bimaculatus*, a peroxidase, and evaluated its antioxidant capacity. Our results show it was effective against ROS. Molecular docking and in vivo SPR techniques were used to evaluate the potential binding properties of TTX to rTbprx-1, which were found to facilitate the entry of tetrodotoxin into cells. We found that Tbprx-1 is a novel tetrodotoxin-binding protein for the first time, and it is highly expressed in immune organs.

## Data Availability

Not applicable.

## References

[1] Sheng Y., Sun Y., Zhang X., Wan H., Yao C., Liang K., Li L., Liu B., Zhong J., Zhang Z. (2020). Characterization of two myostatin genes in pufferfish *Takifugu bimaculatus*: Sequence, genomic structure, and expression. PeerJ.

[2] Shi Y., Zhou Z., Liu B., Kong S., Chen B., Bai H., Li L., Pu F., Xu P.J. (2020). Construction of a high-density genetic linkage map and QTL mapping for growth-related traits in *Takifugu bimaculatus*. Mar. Biotechnol..

[3] Zhou Z., Liu B., Chen B., Shi Y., Pu F., Bai H., Li L., Xu P. (2019). The sequence and de novo assembly of *Takifugu bimaculatus* genome using PacBio and Hi-C technologies. Sci. Data.

[4] Shen H., Li Z., Jiang Y., Pan X., Wu J., Cristofori-Armstrong B., Smith J.J., Chin Y.K.Y., Lei J., Zhou Q. (2018). Structural basis for the modulation of voltage-gated sodium channels by animal toxins. Science.

[5] Lago J., Rodríguez L.P., Blanco L., Vietes J.M., Cabado A.G. (2015). Tetrodotoxin, an Extremely Potent Marine Neurotoxin: Distribution, Toxicity, Origin and Therapeutical Uses. Mar. Drugs.

[6] Miyazawa K., Noguchi T. (2001). Distribution and origin of tetrodotoxin. J. Toxicol. Toxin Rev..

[7] Noguchi T., Arakawa O. (2008). Tetrodotoxin–Distribution and Accumulation in Aquatic Organisms, and Cases of Human Intoxication. Mar. Drugs.

[8] Dong W.R., Xiang L.X., Shao J.Z. (2007). Cloning and characterisation of two natural killer enhancing factor genes (NKEF-A and NKEF-B) in pufferfish, Tetraodon nigroviridis. Fish Shellfish Immunol..

[9] Wood Z.A., Schröder E., Harris J.R., Poole L.B. (2003). Structure, mechanism and regulation of peroxiredoxins. Trends Biochem. Sci..

[10] Li C., Ni D., Song L., Zhao J., Li L. (2008). Molecular cloning and characterization of a catalase gene from Zhikong scallop Chlamys farreri. Fish Shellfish Immunol..

[11] Sharapov M.G., Novoselov V.I., Ravin V.K. (2016). Xenopus laevis peroxiredoxins: Gene expression during development and characterization of the enzymes. Mol. Biol..

[12] Neumann C., Krause D., Carman C., Das S., Dubey D.P., Abraham J.L., Bronson R.T., Fujiwara Y., Orkin S.H., Van Etten R.A. (2003). Essential role for the peroxiredoxin Prdx1 in erythrocyte antioxidant defence and tumour suppression. Nature.

[13] Edgar R.S., Green E.W., Zhao Y., van Ooijen G., Olmedo M., Qin X., Xu Y., Pan M., Valekunja U.K., Feeney K.A. (2012). Peroxiredoxins are conserved markers of circadian rhythms. Nature.

[14] Han Y.H., Zhang Y.Q., Jin M.H., Jin Y.H., Qiu M.Y., Li W.L., He C., Yu L.Y., Hyun J.W., Lee J. (2020). Peroxiredoxin I deficiency increases keratinocyte apoptosis in a skin tumor model via the ROS-p38 MAPK pathway. Biochem. Biophys. Res. Commun..

[15] Matsui T., Yamamori K., Furukawa K., Kono M. (2000). Purification and some properties of a tetrodotoxin binding protein from the blood plasma of kusafugu, *Takifugu niphobles*. Toxicon.

[16] Yotsu-Yamashita M., Sugimoto A., Terakawa T., Shoji Y., Miyazawa T., Yasumoto T. (2001). Purification, characterization, and cDNA cloning of a novel soluble saxitoxin and tetrodotoxin binding protein from plasma of the pufferfish, *Fugu pardalis*. Eur. J. Biochem..

[17] Yotsu-Yamashita M., Yamaki H., Okoshi N., Araki N. (2010). Distribution of homologous proteins to puffer fish saxitoxin and tetrodotoxin binding protein in the plasma of puffer fish and among the tissues of *Fugu pardalis* examined by Western blot analysis. Toxicon.

[18] Matsumoto T., Tanuma D., Tsutsumi K., Jeon J.K., Ishizaki S., Nagashima Y. (2010). Plasma protein binding of tetrodotoxin in the marine puffer fish *Takifugu rubripes*. Toxicon.

[19] Yotsu-Yamashita M., Okoshi N., Watanabe K., Araki N., Yamaki H., Shoji Y., Terakawa T. (2013). Localization of pufferfish saxitoxin and tetrodotoxin binding protein (PSTBP) in the tissues of the pufferfish, *Takifugu pardalis*, analyzed by immunohistochemical staining. Toxicon.

[20] Tatsuno R., Yamaguchi K., Takatani T., Arakawa O. (2013). RT-PCR- and MALDI-TOF mass spectrometry-based identification and discrimination of isoforms homologous to pufferfish saxitoxin- and tetrodotoxin-binding protein in the plasma of non-toxic cultured pufferfish (*Takifugu rubripes*). Biosci. Biotechnol. Biochem..

[21] Hashiguchi Y., Lee J.M., Shiraishi M., Komatsu S., Miki S., Shimasaki Y., Mochioka N., Kusakabe T., Oshima Y. (2015). Charac-terization and evolutionary analysis of tributyltin-binding protein and pufferfish saxitoxin and tetrodotoxin-binding protein genes in toxic and nontoxic pufferfishes. J. Evol. Biol..

[22] Satone H., Nonaka S., Lee J.M., Shimasaki Y., Kusakabe T., Kawabata S.I., Oshima Y. (2017). Tetrodotoxin- and tributyltin-binding abilities of recombinant pufferfish saxitoxin and tetrodotoxin binding proteins of *Takifugu rubripes*. Toxicon.

[23] Yotsu-Yamashita M., Nagaoka Y., Muramoto K., Cho Y., Konoki K. (2018). Pufferfish Saxitoxin and Tetrodotoxin Binding Protein (PSTBP) Analogues in the Blood Plasma of the Pufferfish Arothron nigropunctatus, A. hispidus, A. manilensis, and Chelonodon patoca. Mar. Drugs.

[24] Yin X., Kiriake A., Ohta A., Kitani Y., Ishizaki S., Nagashima Y. (2017). A novel function of vitellogenin subdomain, vWF type D, as a toxin-binding protein in the pufferfish *Takifugu pardalis* ovary. Toxicon.

[25] Qiao K., Jiang C., Xu M., Chen B., Qiu W., Su Y., Hao H., Lin Z., Cai S., Su J. (2021). Molecular Characterization of the Von Willebrand Factor Type D Domain of Vitellogenin from *Takifugu flavidus*. Mar. Drugs.

[26] Qiao K., Fang C., Chen B., Liu Z., Pan N., Peng H., Hao H., Xu M., Wu J., Liu S. (2020). Molecular characterization, purification, and antioxidant activity of recombinant superoxide dismutase from the Pacific abalone *Haliotis discus hannai* Ino. World J. Microbiol. Biotechnol..

[27] Wang L., Guo H., Zhang N., Ma Z., Jiang S., Zhang D. (2015). Molecular characterization and functional analysis of a peroxiredoxin 1 cDNA from golden pompano (*Trachinotus ovatus*). Dev. Comp. Immunol..

[28] Sun C.C., Dong W.R., Shao T., Li J.Y., Zhao J., Nie L., Xiang L.X., Zhu G., Shao J.Z. (2017). Peroxiredoxin 1 (Prx1) is a dual-function enzyme by possessing Cys-independent catalase-like activity. Biochem. J..

[29] Neumann C.A., Cao J., Manevich Y.J. (2009). Peroxiredoxin 1 and its role in cell signaling. Cell Cycle.

[30] Xu K., Wang G. (2018). Identification and characterization of peroxiredoxin 1 from *Lateolabrax japonicus* under biotic and abiotic stresses. Fish Shellfish Immunol..

[31] König J., Lotte K., Plessow R., Brockhinke A., Baier M., Dietz K.J. (2003). Reaction mechanism of plant 2-Cys peroxiredoxin. Role of the C terminus and the quaternary structure. J. Biol. Chem..

[32] Cai L., Wu X., Zhang Y., Li X., Ma S., Li J. (2015). Purification and characterization of three antioxidant peptides from protein hydrolysate of grass carp (*Ctenopharyngodon idella*) skin. J. Funct. Foods.

[33] Shirato K., Takanari J., Ogasawara J., Sakurai T., Imaizumi K., Ohno H., Kizaki T. (2016). Enzyme-treated asparagus extract attenuates hydrogen peroxide-induced matrix metalloproteinase-9 expression in murine skin fibroblast L929 cells. Nat. Prod. Commun..

[34] Madusanka R.K., Neranjan Tharuka M.D., Madhurranga W.S.P., Lee S., Lee J. (2020). Transcriptional modifications and the cytoprotective, DNA protective, and wound healing effects of peroxiredoxin-1 from *Sebastes schlegelii*. Fish Shellfish Immunol..

[35] Heo S., Kim S., Kang D. (2020). The Role of Hydrogen Peroxide and Peroxiredoxins throughout the Cell Cycle. Antioxidants.

[36] Sangpairoj K., Changklungmoa N., Vanichviriyakit R., Sobhon P., Chaithirayanon K. (2014). Analysis of the Expression and Antioxidant Activity of 2-Cys Peroxiredoxin Protein in *Fasciola gigarttica*. Exp. Parasitol..

[37] De Zoysa M., Pushpamali W.A., Whang I., Kim S.J., Lee J. (2008). Mitochondrial thioredoxin-2 from disk abalone (*Haliotis discus discus*): Molecular characterization, tissue expression and DNA protection activity of its recombinant protein. Comp. Biochem. Physiol. Part B Biochem. Mol. Biol..

